# Posttranslational modification and beyond: interplay between histone deacetylase 6 and heat-shock protein 90

**DOI:** 10.1186/s10020-021-00375-3

**Published:** 2021-09-16

**Authors:** Ping Liu, Ji Xiao, Yiliang Wang, Xiaowei Song, Lianzhou Huang, Zhe Ren, Kaio Kitazato, Yifei Wang

**Affiliations:** 1 College of Life Science and Technology, Guangzhou Jinan Biomedicine Research and Development Center, Jinan University, Guangzhou, China; 2 College of Pharmacy, Jinan University, Guangzhou, China; 3 Department of Clinical Research Pharmacy, Graduate School of Biomedical Sciences, Nagasaki University, Nagasaki, Japan

**Keywords:** HDAC6, Hsp90, Client proteins, Acetylation, Drug development

## Abstract

Posttranslational modification (PTM) and regulation of protein stability are crucial to various biological processes. Histone deacetylase 6 (HDAC6), a unique histone deacetylase with two functional catalytic domains (DD1 and DD2) and a ZnF-UBP domain (ubiquitin binding domain, BUZ), regulates a number of biological processes, including gene expression, cell motility, immune response, and the degradation of misfolded proteins. In addition to the deacetylation of histones, other nonhistone proteins have been identified as substrates for HDAC6. Hsp90, a molecular chaperone that is a critical modulator of cell signaling, is one of the lysine deacetylase substrates of HDAC6. Intriguingly, as one of the best-characterized regulators of Hsp90 acetylation, HDAC6 is the client protein of Hsp90. In addition to regulating Hsp90 at the post-translational modification level, HDAC6 also regulates Hsp90 at the gene transcription level. HDAC6 mainly regulates the Hsp90-HSF1 complex through the ZnF-UBP domain, thereby promoting the HSF1 entry into the nucleus and activating gene transcription. The mutual interaction between HDAC6 and Hsp90 plays an important role in the regulation of protein stability, cell migration, apoptosis and other functions. Plenty of of studies have indicated that blocking HDAC6/Hsp90 has a vital regulatory role in multifarious diseases, mainly in cancers. Therefore, developing inhibitors or drugs against HDAC6/Hsp90 becomes a promising development direction. Herein, we review the current knowledge on molecular regulatory mechanisms based on the interaction of HDAC6 and Hsp90 and inhibition of HDAC6 and/or Hsp90 in oncogenesis and progression, antiviral and immune-related diseases and other vital biological processes.

## Background

Post-translational modification (PTM) of proteins is a common form of epigenetic regulation. Acetylation, one of the posttranslational modifications of proteins, plays a powerful role in regulating gene expression, protein stability, and enzyme activity (Morgan and Shilatifard 2020). Typically, acetylation consists of histone and nonhistone acetylation modifications. Acetylation modifications of histone proteins regulate chromatin compaction and mediate the epigenetic regulation of gene transcription (Glozak et al. 2005). Nonhistone acetylation modifications are mainly involved in protein degradation and enzyme activity regulation (Narita et al. 2019). The histone deacetylase (HDAC) family is a large family of primary regulators that participate in histone acetylation. In addition, several HDAC members, especially the cytoplasmic protein HDAC6, play a significant role in the regulation of nonhistone acetylation. HDAC6 is closely related to multiple diseases involved in malignant tumors (Li et al. 2018), pathogen infection (Zhang et al. 2016), cardiovascular and metabolic diseases (Bagchi and Weeks 2019), neurodegenerative diseases and pathological autoimmune responses (Simões-Pires et al. 2013) by regulating the acetylation of nonhistone proteins (Seidel et al. 2015). Specifically, nonhistone proteins have been identified as substrates of HDAC6, including Hsp90, α-tubulin, cortactin, Ku70, RIG-I and β-Catenin (Zheng et al. 2017). Hsp90 is a member of the family of heat shock proteins (HSPs), which perform their molecular chaperone functions to regulate the folding and degradation of client proteins and further affect various biological functions. A large number of studies have reported that HDAC6 is able to deacetylate Hsp90. Simultaneously, HDAC6 is also a client protein of Hsp90, and the protein stability of HDAC6 is modulated by Hsp90.

Due to the significant biological functions of HDAC6 and Hsp90, exploring inhibitors for antitumor and antiviral biological effects has become a high-profile direction for new drug development. Therefore, we review the mutual regulatory pathway, molecular mechanisms, biological functions and related diseases between HDAC6 and Hsp90, as well as research exploring efficient and specific inhibitors or combined moderator applications, providing a reference for comprehensive recognition and an in-depth understanding of the regulatory mechanism between HDAC6 and Hsp90 molecules and innovative drug exploitation.

### HDAC6 is a specific member of the histone deacetylase family

**Fig. 1:**
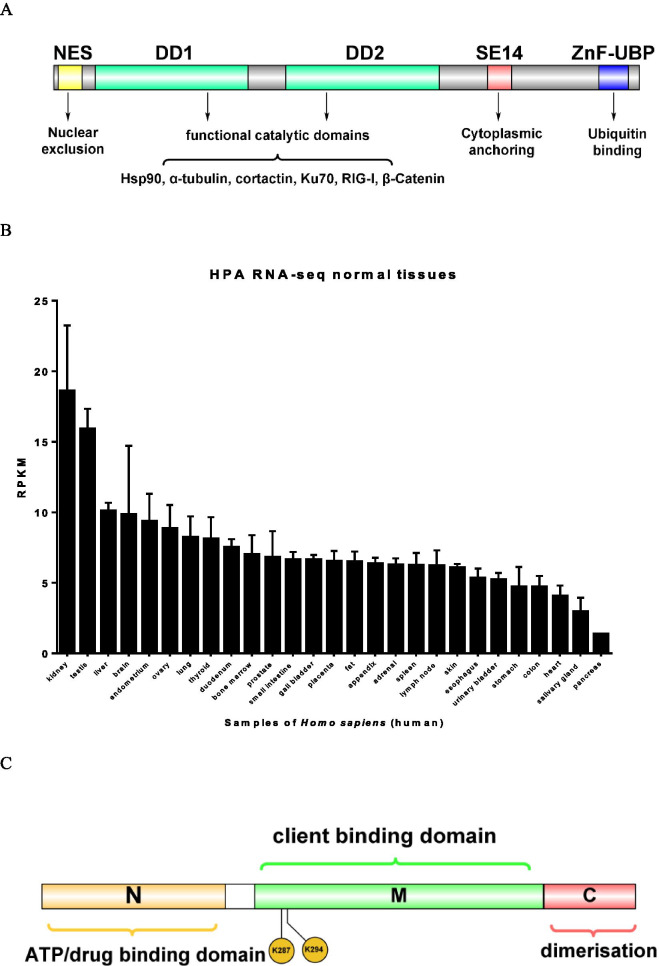
The protein structure diagram of HDAC6 and mRNA expression in different organs of *Homo sapiens*. **A** HDAC6 consists of two functional catalytic domains (DD1 and DD2) and a ZnF-UBP domain (ubiquitin binding domain, BUZ). **B** HDAC6 mRNA is widely expressed in various organs of Homo sapiens. **C** Domain architecture and alternative acetylation site of Hsp90 (N, N-terminal binding region; C, C-terminal binding region; M, intermediate connection region)

In mammals, HDACs have been phylogenetically classified into four classes based on their homology of accessory domains to histone deacetylases. Class I HDACs include HDAC1, HDAC2, HDAC3 and HDAC8. Class II HDACs are HDAC4, HDAC5, HDAC6, HDAC7, HDAC9 and HDAC10, and according to their sequence homology and domain organization, they are further categorized into class IIa (HDAC4, HDAC5, HDAC7 and HDAC9) and class IIb (HDAC6 and HDAC10). Class III HDACs are made up of Sir2-like deacetylase silent information regulators (Sirt1, Sirt2, Sirt3, Sirt4, Sirt5, Sirt6 and Sirt7) (Ruijter et al. 2003). As a distinct member of HDACs in higher eukaryotes, HDAC6 was first discovered in mice (Verdel and Khochbin 1999). HDAC6 presents different molecular features and functions from other HDAC family members based on the molecular structure characteristics of two functional catalytic domains (DD1 and DD2) and a ZnF domain (ubiquitin binding domain, BUZ) (Fig. 1A). In contrast to the nuclear location of other HDAC family members, HDAC6 is a unique deacetylase due to its cytoplasmic localization and more powerful ability to deacetylate proteins than histones and is widely expressed in various normal tissues and organs of *Homo sapiens* (humans) (Fig. 1B) (Data from https://www.ncbi.nlm.nih.gov/gene/10013). HDAC6 contains a noncatalytically active ubiquitin-bound zinc finger structure (called the ZnF-UBP domain) and a dynamin-binding domain at the carboxyl terminus. HDAC6 deacetylates nonhistone substrates, including α-tubulin, Hsp90, cortactin, Ku70, and β-Catenin (Hubbert et al. 2002; Matsuyama et al. 2002; Selenica et al. 2014). HDAC6 was previously shown to be a pivotal element in the misfolded protein accumulation-induced stress response by coordinating the clearance of protein aggregates through aggresome formation and their autophagic degradation (Boyault et al. 2007; Matthias et al. 2008). Together with the motor protein dynein, HDAC6 carries cytotoxic polyubiquitinylated proteins into autophagosomes (Li et al. 2013). Therefore, the two functional domains perform important but different functions.

The ubiquitination domain (ZnF-UBP) of HDAC6 is conducive to the formation of pressure particle SG, which can be prevented by interfering with the arrangement of microtubules or damaging dynamin. As an antiviral immune complex, stress particle SGs play positive roles in the IFN immune response and can be autophagy targeted. HDAC6 can mediate SG degradation after being recognized by P62/SQSTM1 (Zheng et al. 2020). In addition, due to the specific ubiquitin binding domain, HDAC6 plays a significant role at the intersection of two important signaling pathways in cells: the protein lysine acetylation pathway and the ubiquitin pathway (Boyault et al. 2007). Because of this, HDAC6 can coordinate and respond to intracellular events under different stress stimuli, including the endoplasmic reticulum stress response (ERS) and the unfolded protein response (UPR) (Iwata et al. 2005; Rao et al. 2008). In response to endoplasmic reticulum stress and unfolded proteins, HDAC6 plays a vital role as a coordinating factor in eliminating toxicity caused by misfolded or unfolded proteins (Li et al. 2017). In addition, HDAC6 plays a key role in the regulation of different cellular stress responses, the results indicate that HDAC6 plays a major regulatory role in different kinds of cellular stress responses. However, the mechanism of HDAC6 in different kinds of cellular stress responses remains unclear (Ryu et al. 2017a).

The ubiquitin–proteasome system (UPS) and autophagy are two important protein degradation pathways in cells. A coordinated and complementary relationship between these two systems is essential for cells to protect themselves from stress. Among them, some important protein regulatory factors, including P62 and HDAC6, play an important role in coordinating with their relationship. The UPS and autophagy are considered key factors in neurodegenerative diseases. Pandy et al. used a Drosophila neurodegenerative disease model to research the relationship between autophagy and HDAC6 and found that HDAC6 autophagy is dependent on the inhibition of the ubiquitin–proteasome pathway, which further reveals the complementary relationship between these two degradation pathways (Zheng and Wang 2010; Pandey et al. 2007). Since the accumulation of protein aggregates is a distinct feature of neurodegenerative diseases, the aggregate-autophagy pathway is expected to be a potential target for the treatment of these diseases (Yan 2014). Notably, the deacetylase activity of HDAC6 is necessary for aggregate formation and can regulate aggregate formation by interacting with other proteins. For example, HDAC6 directly interacts with P62 to regulate aggregate formation, which is localized in ubiquitinated protein aggregates (Yan et al. 2013). However, not all HDAC6 ubiquitin domain-mediated autophagy is associated with its deacetylase activity. HR23B is a biomarker in response to HDAC inhibitors. In the process of the interaction, HR23B expression level determines different cell fates. HDAC inhibitors can lead to apoptosis in cells which expressing high levels of HR23B; In contrast, when HR23B at a low expression level in cells, an HDAC inhibitor promotes autophagy. The specific mechanism of the ZnF-UBP domain of HDAC6 on aggregates and autophagy is as follows: HDAC6 binds to the misfolded proteins of ubiquitination, transports them along the microtubule skeleton to the cell motor protein, transports them to the aggregates, and finally degrades through the lysosomal-autophagy pathway (New et al. 2013; Kalveram et al. 2008). The elucidating of this mechanism will help to enhance the understanding of the molecular mechanism of cancer and provide theoretical basis and unique insights for further exploration of new cancer therapies.

HDAC6 is widely involved in the treatment of various human diseases in the form of inhibitors. HDAC6 inhibitors are gradually being used in clinical drug development and are expected to be a new cancer treatment strategy. For example, Benoy et al. developed three HDAC6 selective inhibitors, ACY-738, ACY-775, and ACY-1215, which have been proven to be effective. These inhibitors have been shown to improve the function of neuromuscular junctions and sensory nerve conduction in gastrocnemius neuromuscle. Notably, ACY-1215 is currently in clinical trials with patients with cancer. This also suggests that inhibition of HDAC6 is a potential strategy for the treatment of CMT2 in axons gastrocnemius (Benoy et al. 2017). In addition, some studies have shown that HDAC6 inhibitors can treat hematological malignancies, which raises the possibility of HDAC inhibitors being administered as potential antitumor drugs. It has been reported that HDAC6 inhibitors have been used in preclinical and clinical studies of lymphoproliferative diseases and have shown certain activity (Cosenza and Pozzi 2018). Currently, more than a dozen HDAC6 inhibitors have been applied in the clinical treatment of cancer, which also proves that HDAC6 is a potential target molecule in clinical drug development.

### Characterization of Hsp90

Heat shock proteins (HSPs) are highly conserved intracellular proteins in eukaryotic cells and prokaryotic cells. They protect cells mainly by regulating the three-dimensional structure of proteins and preventing misfolding and degradation of proteins by the proteasome (Calderwood et al. 2007). HSPs is abundant in cells, accounting for 1%–2% of the total protein, and when subjected to heat, oxidation, chemical stimulation and other stress states, the expression of heat shock proteins will be up-regulated, accounting for about 4%–6% of the total cell proteins (Choudhary et al. 2009). According to the molecular weight, Hsp protein families are mainly divided into five categories, namely small molecule heat shock proteins (12–43KDa), Hsp60, Hsp70 (66–78KDa), Hsp90 (83–94KDa) and Hsp100, among which Hsp60, Hsp70 and Hsp90 are the most widely studied (Lebret et al. 2003). Hsp27 is located in the cytoplasm and its function is mainly to prevent the aggregation of unfolded proteins. Mitochondrial protein Hsp60 is mainly involved in mitochondrial protein folding and assembly. Hsp70 is distributed in mitochondria, nucleus and cytoplasm, and participates in protein transport, anti-apoptosis, and protein degradation (Sreedharan and Why 2016). Hsp90 is mainly composed of four parts: N-terminal binding region, connecting region connecting N-terminal and middle region, middle region and C-terminal binding region (Fig. 1C). In mammalian cells, there are two or more genes encoding cytosolic Hsp90 homologs (Chen et al. 2006). HSP90A is a cytosolic family protein that includes Hsp90α and Hsp90β, which are inducible and constitutively expressed forms, respectively (Chen et al. 2005). As an ATP-dependent molecular chaperone, Hsp90 plays a crucial adjuvant role in the process of cellular protein synthesis, correct folding, configuration stability, assembly, transportation and degradation.

According to the role of molecular chaperones in protein folding, there are three main folding pathways: (1) spontaneous chaperone-independent folding; (2) folding assisted by repetitive Hsp70 cycles; and (3) folding by Hsp70-Hsp90 cascades. Among them, helper chaperones such as STI1/Hop play an important role in the Hsp70-Hsp90 chaperone cascade, which promotes the interaction between Hsp70 and Hsp90 by binding their C-termini. Most importantly, Hsp90 has two distinct functions in protein folding: an older, evolutionarily conserved function in protein folding downstream of HSP70 that is independent of the helper chaperone. Second, in the complex and intricate signal network regulation of eukaryotic cytoplasm, it is precisely regulated by many cochaperones (Morán Luengo et al. 2019). Advances in the mechanisms of molecular chaperones and the development of Hsp90 inhibitors may be conducive to stimulate a new generation of therapies that are critical not only for cancer but also for other protein misfolding diseases such as neurodegenerative diseases.

In addition to being molecular chaperones, heat shock proteins also play crucial roles in the activation of the heat shock response. The unfolded and/or misfolded proteins that have accumulated due to cellular stress can be replaced by activation of the heat shock response (HSR). Since Hsp90 is the main regulator of HSR, the regulation of Hsp90 by small molecules represents a therapeutic approach to complications of the peripheral nervous system and central neuropathy (Chaudhury et al. 2021). In addition, Hsp90 plays an important role in a variety of human diseases, such as cancer, inflammation, Alzheimer's disease, Parkinson's disease, and diseases related to protein misfolding. Therefore, Hsp90 is expected to become a new target molecule for drug development, and many of its client proteins are being used as targets for carcinoma therapy in clinical practice (Table 1). However, rather than using the client protein of Hsp90 as a target molecule, it would be better to use Hsp90 as a direct target for tumor therapy, thus achieving targeted therapy. Clinical studies have shown that Hsp90 is significantly positive in patients with early nasopharyngeal carcinoma, and the mechanism may be related to the effect of Hsp90 on the growth and proliferation of human nasopharyngeal carcinoma cells, suggesting that Hsp90 can be used as a marker for early screening of nasopharyngeal carcinoma (Liu et al. 2019). 17-DMAG is a semisynthetic derivative of 17-AAG, and the class I clinical drug Hsp90 has entered clinical trials and can be used alone or in combination with other effective anticancer drugs to optimize the anticancer effect. It is widely used in the treatment of leukemia, melanoma, breast cancer, lymphoma, liver cancer, lung cancer, multiple myeloma and other diseases (Mellatyar et al. 2018). Currently, an increasing number of studies have reported the development of Hsp90-targeted drugs with high specificity, strong potency and similar drug properties, indicating that they are very likely to have important clinical application value (Banerjee et al. 2020).

**Table 1:** Effector and processing based on HDAC6 deacetylation activity of Hsp90

Effector or client protein of Hsp90	Biological process or pathway	Intervention method of HDAC6	References
GR	Glucocorticoid receptor maturation in A431, A549 cells or serotonin pathways in mice	Mutations, trichostatin A (TSA) (1 μM), Tubacin (10 μM), ACY-738(2.5 μM) knock out or transgenesis	Espallergues et al. (2012); Hu et al. (2019); Jochems et al. (2015); Kovacs et al. (2005a, b); Lee et al. (2012); Murphy et al. (2005); Zhu et al. (2020)
ERα	Hippo pathway in ERα + or tamoxifen-resistant breast cancer	Tubacin (1 μM), Panobinostat (LBH589) (100 nM) or Dacinostat (LAQ824) (250 nM)	Fiskus et al. (2007); Yu et al. (2017)
HIF-1α	Glioblastoma growth and angiogenesis	LBH589 (40 nM) or TSA (500 nM)	Schoepflin et al. (2016); Yao et al. (2017); Zhang et al. (2010)
EGFR	Esophageal squamous cell carcinoma proliferation and migration	siRNA or Tubastatin (1 μM)	Tao et al. (2018); Kim et al. (2009); Gravina et al. (2013)
Kit	Growth inhibition, cell cycle arrest, apoptosis against malignant mast cell lines	AR-42 (3 μM)	Lin et al. (2010)
Her-2	Her-2 stability via the proteasome pathway in SK-BR-3 cells	Carbamazepine (CBZ) (100 μM) or TSA) (100 nM)	Kim et al. (2009); Gravina et al. (2013); Meng et al. (2011)
VEGFRs	Proteasomal degradation of vascular endothelial growth factor receptors	Suberoylanilide hydroxamic acid (SAHA) (1 μM), LAQ824 (300 nM) or TSA (30 nM) or Tubacin (100 nM)	Park et al. (2008); Verheyen et al. (2012)
AR	Proteasome degradation of AR in LNCaP cells or prostate cancer	siRNA, overexpression, genistein (25 μM), 4-HBAs (4-hydroxybenzoic acids) (50 μM), SAHA (10 μM), Sulforaphane (20 μM), Belinostat (PXD101) (0.5 μM) or 2‐75 (2.5 µM)	Hu et al. (2019); Gravina et al. (2013); Ai et al. (2009); Basak et al. (2008); Gibbs et al. (2009); Rosati et al. (2016); Seidel et al. (2016)
AhR	Aryl hydrocarbon receptor signaling in human aerodigestive epithelial cells	TSA (500 nM), SAHA (20 μM) or Tubacin (20 μM)	Kekatpure et al. (2009)
Rac1	Membrane ruffle formation and cell migration in MEFs	knock-out HDAC6	Gao et al. (2007)
Survivin	Autophagy and viability reduction in breast cancer cells	SAHA (1.4 μM)	Lee et al. (2016)
Bcr-Abl	Degradation of Bcr-Abl in human leukemia cells or FDCP-1 cells	LAQ824(100 nM), SAHA (5 μM), MRLB-223(10 μM) or siRNA	Rao et al. (2008); Bali et al. (2005); Lee et al. (2007); Newbold et al. (2013)
c-Raf	Degradation of c-Raf human in leukemia cells	Tubacin (3 µM) and siRNA	Rao et al. (2008)
tau	Degradation of tau in HEK293T or HeLa cells and tau pathology neurodegeneration in rTg4510 mouse models	Overexpression HDAC6, siRNA or tubastatin (25 mg/kg/day)	Selenica et al. (2014); Cook et al. (2012)
aurora-A	Migration and metastatic activity in breast cancer	MPT0G211 (10 μM) or tubastatin A (10 μM)	Hsieh et al. (2019)
IKKα	Proliferation and apoptosis in human myeloma cell line and primary myeloma cells	As_2_O_3_(5 μM)	Qu et al. (2012)
MIF	MIF stability via the proteasome pathway in cancer cells	siRNA or SAHA (5 μM)	Schulz et al. (2012)
cyclin D1 and CDK4	G1 growth arrest in lung cancer cells	NBM-T-BBX-OS01 (10 μM) or overexpression	Pai et al. (2015)
mutant p53	Preferential cytotoxicity for mutp53 tumor	SAHA (5 μM), siRNA, or A452(2 μM)	Li et al. (2011); Ryu et al. (2017b)
MMP-9	Migration and invasion activities in lung cancer	Honokiol (10 μM)	Pai et al. (2020)
NS5(Japanese Encephalitis Virus)	Viral RNA Synthesis	Tubacin (10 μM) or TBSA (10 μM)	Lu et al. (2017)

### HDAC6 modulates Hsp90 client proteins by deacetylase activity

**Fig. 2:**
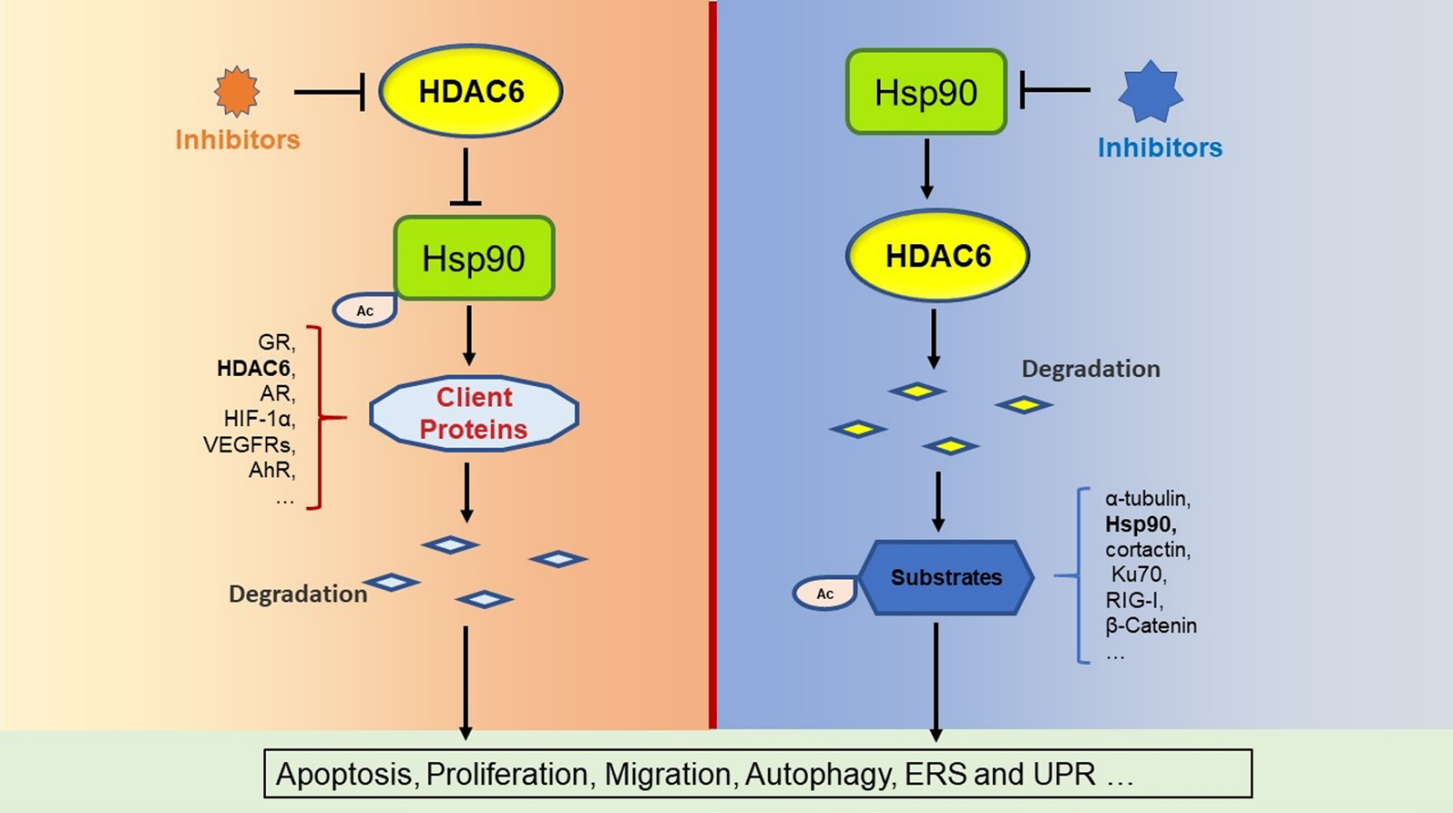
Interplay between HDAC6 and Hsp90

The biological activity of Hsp90 is regulated by multiple pathways including acetylation. The acetylation sites mapped to K294 (K295 in the mouse ortholog protein) and K287 in Hsp90α and Hsp90β, respectively (Scroggins et al. 2007) (Fig. 1C). Acetylation at K69 or K294 reduces ATP binding to Hsp90 and decreases Hsp90 binding to its cochaperones and client proteins. HDAC6 and Hsp90 are present in a cytoplasmic complex, and HDAC6 inhibition leads to Hsp90 acetylation and disruption of its key chaperone functions. HDAC6, which is the deacetylase of Hsp90, thereby leading to hyperacetylation of Hsp90, specifically impairs the chaperone function of Hsp90 by decreasing the affinity of Hsp90 for ATP and client proteins and thus promoting the polyubiquitination and subsequent degradation of Hsp90 client proteins, such as GR, ERα, AR, HIF-1α, VEGFRs, AhR, Her2, Racl, and EGFR (Fig. 2). HDAC6 also controls mitochondrial metabolic activity partly through deacetylation of Hsp90 (Kamemura et al. 2012). In addition, some studies have shown that hindering HDAC6 can not only induce the acetylation modification of Hsp90 but can also directly cause Hsp90 fragmentation and then enhance microglial activation and migration (Tsai et al. 2015; Gao et al. 2007). In view of the large number of client proteins of Hsp90, studies have directly aimed to suppress Hsp90 or affect the function of Hsp90 by regulating HDAC6. In addition to the straightforward regulatory signal pathways and biological function changes of interest, the significant and valuable Hsp90 regulation process that impacts various biological phenomena also needs to be closely monitored to ensure that the discovery of biological phenomena is one of the main ways of regulating Hsp90.

### HDAC6 is regulated by Hsp90 as a client protein

As mentioned above, HDAC6 can acetylate Hsp90 and affect the stability of its client proteins. Interestingly, several lines of evidence indicate that HDAC6 is one of the client proteins of Hsp90 (Fig. 2). HDAC6 protein degradation is regulated by the Hsp90 chaperone function in turn in K562 cells and primary acute myeloid leukemia (AML) (Rao et al. 2008). AT13387, a ground-breaking Hsp90 inhibitor, can restrain Hsp90 molecular chaperone function, downregulate HDAC6 and then increase the acetylation and stabilization of α-tubulin, ultimately suppressing nasopharyngeal carcinoma (NPC) oncogenesis and progression (Chan et al. 2013). In conclusion, there is a mutual regulatory relationship between HDAC6 and HSP90, and a thorough understanding of their regulatory mechanism will be helpful for the further development of new clinical drug therapy.

### HDAC6 regulates Hsp90 by its ZnF-UBP

**Fig. 3:**
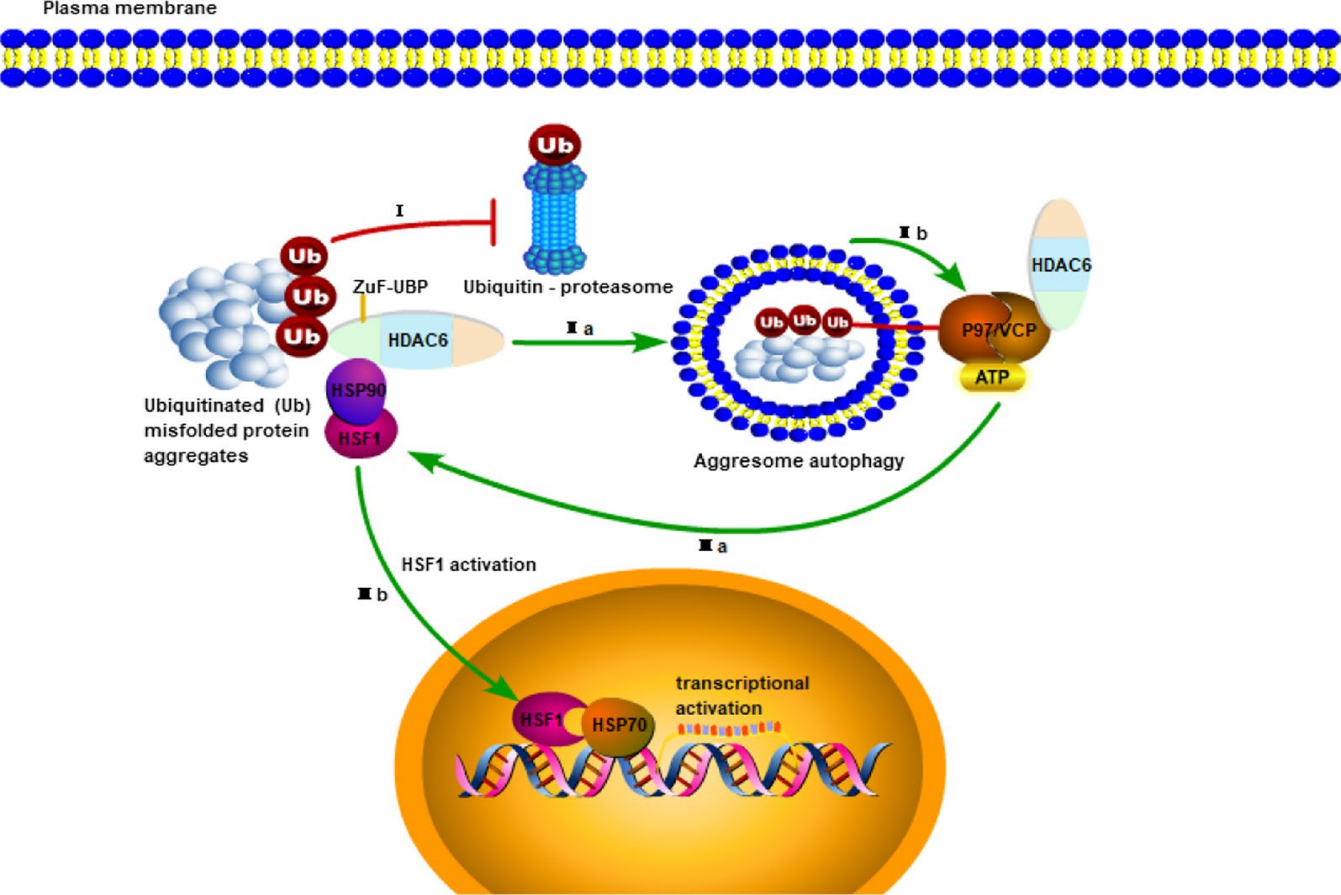
Regulation Hsp90 by the ZnF-UBP of HDAC6

The ZnF-UBP domain of HDAC6 interacts with a variety of proteins, enzymes and molecular chaperones and plays a key regulatory role in a variety of human diseases. The E3 ubiquitin ligase TRIM50 promotes the formation and degradation of ubiquitinated proteins through its interaction with HDAC6 (Fusco et al. 2012), and the Cullin3 (SPP) ubiquitin E3 ligase has also been shown to promote HDAC6 polyubiquitination and degradation (Tan et al. 2017). HDAC6 has been shown to modulate radiosensitivity in NSCLC by promoting CHK1 degradation (Moses et al. 2020). Ubiquitin ligase Parkin mutation, which causes early onset familial Parkinson's disease, catalyzes mitochondrial ubiquitination and promotes mitochondrial binding to HDAC6 and P62, leads to mitochondrial clearance (Lee et al. 2010). HDAC6 also regulates Hsp90 through the ZnF-UBP domain. The main mechanism is the binding of ubiquitinated protein aggregates through the ubiquitin-binding functional domain of HDAC6. The binding of HDAC6 to ubiquitin leads to the dissociation of the HDAC6-P97/VCP complex, which further dissociates the Hsp90-HSF1 complex through the ATPase activity of HDAC6, and then HSF1 is activated (Boyault et al. 2007) (Fig. 3). HDAC6 protects cells from cytotoxic effects caused by abnormal protein folding and is crucial in biomedicine and in human disease, and in healthy development, it plays an irreplaceable role. It seems that the combined inhibition may lead to impressive therapeutic benefits based on the HDAC6-Hsp90 relationship.

### Development and application of HDAC6 and Hsp90 inhibitors based on their relationship

HDAC6 and Hsp90 are widely investigated anticancer drug targets. Current data indicate that there has an extremely close interaction between HDAC6 and Hsp90 (Fig. 2). Their interdependence and participation in overlapping signaling networks make them ideal candidates for multitargeting approaches, particularly in cancer cells (Krämer et al. 2014). To date, there have been numerous reports to prove the effectiveness of the interaction between the two in clinical application. For example, Park, Younghee et al. demonstrated the prognostic value of the association between HDAC6 and Hsp90 in patients with early-stage breast cancer and proposed a new approach to target HDAC6 and Hsp90 in the clinical treatment of breast cancer (Park et al. 2015). In addition, several studies have reported the role of HDAC6 and Hsp90 in the treatment of prostate cancer. Basak, Shashwati et al. demonstrated a new mechanism by which genistein downregulates the androgen receptor AR protein by inhibiting the chaperone function of HDAC6-Hsp90. This suggests that genistein may be used as a potential chemoprophylaxis for prostate cancer in combination with known inhibitors of HDAC6 and Hsp90 (Basak et al. 2008). HDAC6 regulates androgen receptor hypersensitivity and nuclear localization by regulating Hsp90 acetylation in castration-resistant prostate cancer, and targeting HDAC6 or in combination with other therapeutic approaches is a promising new strategy for the prevention and/or treatment of castration-resistant prostate cancer (Ai et al. 2009). It is also possible to optimize treatment by designing dual-acting compounds of AR and HDAC6 (Jadhavar et al. 2016). Inhibition of HDAC6 enhanced the binding of Hsp90 to 17-AAG in human leukemia cells (Rao et al. 2008). Therefore, combined with the information presented herein, exploring original and high-efficiency drugs or combined utilization targeting HDAC6 and/or Hsp90 is a hot research direction, especially for antitumor applications (Tao et al. 2018; Rosati et al. 2016; Seidel et al. 2016; Qu et al. 2012). Luca Pinzi developed and applied an integrated computational strategy to design dual inhibitors of HDAC6 and Hsp90, and some of them were able to selectively provide an increased level of acetylation of α-tubulin; notably, two compounds also demonstrated a reduction in breast cancer cell proliferation (Pinzi et al. 2020). Significant progress has also been made in the development of efficient and specific moderators of HDAC6, taking advantage of the latest and powerful protein degradation method PROTACs (An et al. 2019). In addition, the combination of inhibitors is also a noteworthy research direction (Yu et al. 2017). A phase I-II study of the histone deacetylase inhibitor vorinostat plus sequential weekly paclitaxel was undertaken for locally advanced breast cancer (Tu et al. 2014). In addition, excavating bispecific inhibitors is continuing. Ritu Ojha synthesized a series of 1-arylpyridine-hydroxamic acids, and their protein inhibitory and antitumor biological activities were evaluated. Compound 12(N1-(1-(2,4-dihydroxy-5-isopropylbenzoyl)indolin-5-yl)-N8-hydroxyoctanediamide) showed significant inhibitory effects on both Hsp90 and HDAC6, as well as significant tumor cytotoxic effects (Ojha et al. 2018). Simultaneously, a series of 4,5,6,7-tetrahydro-isoxazolo-[4,5-c]-pyridines were synthesized, and most of them showed inhibitory activity against Hsp90. Moreover, a derivative bearing a hydroxamic acid residue bound to the C-3 amide portion was found to inhibit both Hsp90 and HDAC6 (Baruchello et al. 2014). The aforementioned studies indicate that the search for dual inhibitors of Hsp90 and HDAC6 is a promising new direction for the development of novel antitumor drugs.

## Conclusions

In this paper, we mainly reviewed the structural characteristics and functions of HDAC6 and Hsp90, as well as the molecular mechanism of their interaction and the latest research progress, with the purpose of providing some insights into clinical drug development of HDAC6 and Hsp90. The interaction between HDAC6 and Hsp90 is closely related to the treatment of human malignant diseases, and the most common treatment is combined therapy with their specific inhibitors. Tremendous progress in basic research and clinical drug development and application has led to significant technological breakthroughs in major diseases such as tumors, viral infections, and imbalances in immune regulation.

As mentioned above, HDAC6-specific inhibitors are widely used in multiple myeloma, studies have shown that As_2_O_3_ plays an anti-multiple myeloma role by inhibiting the activity of HDAC6, promoting the acetylation of α-tubulin, reducing the function of Hsp90, and leading to the inactivation of NF-κB. This result provides an important insight into the molecular mechanism of As_2_O_3_'s anti-myeloma activity, and provides theoretical support for HDAC6 to serve for clinical diseases. However, the study did not have clinical samples to support its conclusions (Qu et al. 2012). In addition, HDAC6-specific inhibitors also used in neurodegenerative disease, the absence of a novel CHIP substrate, HDAC6, has been shown to mitigate the abnormal accumulation of Tau, while the absence of HDAC6 activity further enhances the efficacy of Hsp90 inhibitors, leading to tau degradation. The results suggest that HDAC6 is a key factor in regulating tau levels and suggest a multifaceted approach to treating neurodegenerative diseases caused by abnormal tau accumulation. However, the specific molecular mechanism of HDAC6-HSP90-Tau in this paper remains unknow. If clinical samples are available, the root will be convincing (Cook et al. 2012). Furthermore, HDAC6 inhibitors have potential application value in the treatment of glioblastoma, Zong-Yang Li et al. confirmed for the first time that ER stress-tolerance (ERST) occurred in Temozolomide (TMZ) resistant glioma cells. In addition, they found that the HDAC6 inhibitor Tubastatin A (TUB) may overcome ERST through two cellular signaling pathways: enhancing the P97/VCP-mediated ubiquitin–proteasome degradation system, and inhibiting the HDAC6-mediated autophagy pathway. The study demonstrates that HDAC6 inhibitors are a potential target for the treatment of gliomas, and demonstrates that the combination of TMZ and TUB may be an effective strategy to facilitate the development of new clinical therapies. However, the authors should assist in verifying the results by directly knocking out the ubiquitin-binding domain of HDAC6 (Zheng and Wang 2010). Last but not least, HDAC6 has been widely reported in cancer therapy (Li et al. 2018). Hsp90 has become a potential target for the treatment of a variety of diseases, including Alzheimer's disease and Parkinson's disease. Although a large number of studies have been carried out in this field, the specific molecular mechanism of their action is still unclear. Therefore, it is very important to develop innovative, specific and efficient Hsp90 inhibitor molecules for clinical application (Alam et al. 2017). In conclusion, various studies using HDAC6 inhibitors alone or in combination with other drugs provide a strong scientific basis for the clinical development of these new drugs in human diseases.

To date, limited by low efficacy, water solubility, and lipid solubility, toxicity, or acquired drug resistance, none of the Hsp90 inhibitors have received clinical approval (Oh et al. 2021). The ever-growing studies reporting the interaction between HDAC6 and Hsp90 and overlapping networks are helpful to further understand the molecular regulation mechanism. Since HDAC6 and Hsp90 have vital functions in the regulation of multifarious signaling pathway, gene expression, protein stability and other cellular functions, the development and research of HDAC6-Hsp90 inhibitors have gradually increased, and some inhibitors have entered the clinical research stage, although the two targets share rare homology. However, this is not enough, and we must seek solutions and develop strategies from the commanding height of innovation, such as the use of nanomaterial delivery inhibitors to solve the problems of water solubility and cytotoxicity to achieve the purpose of safety and efficiency. On the basis of a full understanding of the specific mechanism and regulatory relationship between their interaction, suitably matched and effective dual inhibitors will be designed and synthesized to create clinical treatments.
